# Impact of the 10-valent pneumococcal conjugate vaccine on antimicrobial prescriptions in young children: a whole population study

**DOI:** 10.1186/s12879-018-3416-y

**Published:** 2018-10-04

**Authors:** Elias Eythorsson, Samuel Sigurdsson, Birgir Hrafnkelsson, Helga Erlendsdóttir, Ásgeir Haraldsson, Karl G Kristinsson

**Affiliations:** 1 University of Iceland, Faculty of Medicine, 101 Reykjavík, Iceland; 2 Department of Mathematics, University of Iceland, Reykjavík, Iceland; 3 Department of Clinical Microbiology, Landspítali University Hospital, 101 Reykjavík, Iceland; 4 Children’s Hospital Iceland, Landspítali University Hospital, Reykjavík, Iceland

**Keywords:** Pneumococcal vaccines, Antibiotic agents, Otitis media, Observational study, Survival analysis

## Abstract

**Background:**

Antimicrobial resistance is a public-health threat and antimicrobial consumption is the main contributor. The ten-valent pneumococcal conjugate vaccine (PHiD-CV10) was introduced into the Icelandic vaccination program in 2011.

The aim was to estimate the vaccine impact of PHiD-CV10 on outpatient antimicrobial prescriptions in children.

**Methods:**

Eleven Icelandic birth-cohorts (2005–2015) were followed from birth until three years of age or to the end of the study period (December 31, 2016). Birth-cohorts were grouped as vaccine non-eligible (VNEC, 2005–2010) or vaccine eligible (VEC, 2011–2015). Data on primary care visits for respiratory infections and antimicrobial prescriptions were extracted from two national registers. Using national identification numbers, prescriptions were linked to physician visits if filled within three days of the visit. Incidence rates and incidence rate ratios between VNEC and VEC were calculated. An Andersen-Gill model was used to model the individual level data, accounting for repeated events and censoring. Vaccine impact was calculated as (1 – Hazard Ratio) × 100%.

**Results:**

Included were 53,510 children who contributed 151,992 person-years of follow-up and filled 231,660 antimicrobial prescriptions. The incidence rate was significantly lower in the VEC compared to the VNEC, 144.5 and 157.2 prescriptions per 100 person-years respectively (IRR 0.92, 95%CI 0.91–0.93). Children in VEC were more likely to have filled zero (IRR 1.16 (95%CI 1.10–1.23) and 1–4 (IRR 1.08 95%CI 1.06–1.11) prescriptions compared to children in VNEC. The vaccine impact of PHiD-CV10 against all-cause antimicrobial prescriptions was 5.8% (95%CI 1.6–9.8%).When only considering acute otitis media-associated prescriptions, the vaccine impact was 21.8% (95%CI 11.5–30.9%).

**Conclusion:**

The introduction of PHiD-CV10 lead to reduced antimicrobial use in children, mainly by reducing acute otitis media episodes. This intervention therefore reduces both disease burden and could slow the spread of antimicrobial resistance.

**Electronic supplementary material:**

The online version of this article (10.1186/s12879-018-3416-y) contains supplementary material, which is available to authorized users.

## Background

Acute otitis media (AOM) and other upper respiratory tract infections (URTI) are the most common causes of ambulatory physician visits and antimicrobial prescriptions in children [1, 2]. The most common bacterial causes of URTI in young children are *Streptococcus pneumoniae* and *Haemophilus influenzae*, though most are caused by viral pathogens [3–6]. Distinguishing between viral and bacterial URTI can be difficult. As much as 30–50% of outpatient antimicrobial prescriptions for URTI are inappropriate [2, 7, 8]. Physicians cite the uncertainty of diagnosis and fear of disease complications as two important factors contributing to inappropriate prescriptions [2, 9–11], which in turn increases antimicrobial resistance [12, 13].

Antimicrobials were once universally recommended in the treatment of AOM to prevent complications. However, when several studies showed no increase in complications in populations with lower prescription rates [2, 14], this endorsement came under scrutiny. As a result, guidelines which recommended more restrictive prescription practices to combat rising antimicrobial resistance were adopted in many countries in the early 2000s [14–17]. This has led to a subsequent decrease in antimicrobial consumption [14, 16, 18, 19].

Concurrently, pneumococcal conjugate vaccines (PCV) were widely introduced into paediatric vaccination programs and have been associated with a decrease in AOM incidence [1, 18, 20–23]. Several randomized controlled trials (RCT) of PCVs have also shown a decrease in both all-cause and URTI-associated antimicrobial prescription rates [24–26].

The 10-valent pneumococcal non-typeable *Haemophilus influenzae* protein D-conjugate vaccine (PHiD-CV10, Synflorix™) was introduced into the Icelandic paediatric vaccination program, with a 2 + 1 schedule without a catch-up for all children born in 2011 and later. Following the introduction, over 97% of each vaccine eligible birth-cohort received ≥2 doses of the vaccine [27]. Prior to the introduction of PHiD-CV10, few children were vaccinated, 0–1.9% of birth-cohorts, while 6.9% and 18.7% of children in birth-cohorts 2009 and 2010 received ≥2 doses [27].

The aim of this study was to investigate the impact of PHiD-CV10 against outpatient antimicrobial prescriptions for children < 3 years of age in Iceland.

## Methods

### Data sources

The study is a whole population observational cohort study of all children under three years of age in Iceland. Eleven consecutive birth-cohorts 2005–2015 were followed until three years of age or to the end of the study period, December 31, 2016. Individual level data on immigration and emigration was obtained from Statistics Iceland. Children who immigrated to Iceland after birth were excluded from the analysis. Observations were censored on death or emigration from Iceland. The 2016 birth-cohort was not included due to short follow-up time.

Data on outpatient antimicrobial prescriptions was extracted from the National Prescription Database (NPD) of the Directorate of Health. The NPD receives automated electronic data from all pharmacies in Iceland, and registers detailed information on each outpatient prescription in the country, identifiable down to the individual and day of purchase. All drug prescriptions with the anatomical therapeutic chemical (ATC) classification code J01 (antibacterials for systemic use) and subgroups were extracted from the NPD.

Information regarding primary care visits for respiratory tract infections was obtained from the Primary Care Database of the Directorate of Health. All visits with International Classification of Diseases, 10th Revision (ICD-10) discharge diagnoses compatible with respiratory tract infections were extracted as previously described [23]. This data was linked to the NPD using national identification numbers. Prescriptions were paired with a physician visit if filled for the same child within three days of the visit. Data from the Primary Care Database was available only through December 31, 2015, restricting the use of linked data to that date. Information on population demographics was acquired from Statistics Iceland (https://www.statice.is/).

### Statistical analysis

The study analysis was done by comparing birth-cohorts individually or grouped by vaccine eligibility. Individual birth-cohorts were each compared to the last vaccine non-eligible birth-cohort (2010) which was used as a reference cohort. Birth-cohorts 2011–2015 were grouped as the vaccine-eligible cohorts (VEC) and birth-cohorts 2005–2010 as the vaccine non-eligible cohorts (VNEC).

Aggregate analyses by calendar year were performed for all children under three years of age. Antimicrobials were classified into six classes; first and second line penicillins, first and second generation macrolides, cephalosporins and others (Table 1) [28]. The proportion of antimicrobial prescriptions in each class was calculated by calendar year. Similarly, the proportion of primary care visits resulting in antimicrobial prescription in each calendar year was calculated by indication. Five groups were defined based on ICD-10 discharge diagnosis; Acute upper respiratory infections (J00-J06), Influenza and pneumonia (J09-J18), Other acute lower respiratory infections (J20-J22), AOM (H65, H66 and H72) and Other viral infections (B34).

**Table 1:** Classification of antimicrobials used in this study

First-line penicillins	Amoxicillin, phenoxymethylpenicillin
Second-line penicillins	Amoxicillin and enzyme inhibitor
First-generation macrolide	Erythromycin
Second-generation macrolide	Azithromycin, clarithromycin,
Cephalosporin	Cefalexin
Others	Ciprofloxacin, clindamycin, pivmecillinam, Trimethoprim/sulfmethoxazole,

### Crude incidence analysis

Incidence rates (IR) per 100 person-years at risk for each of the study birth-cohorts were calculated in six-month age-groups and 95% confidence intervals estimated using the Wald method. Incidence rate ratios (IRR) between the VNEC and the VEC were estimated and 95% confidence intervals calculated assuming Poisson variance.

The cumulative proportion of outpatient antimicrobial prescriptions by three years of age was compared between VNEC and VEC using the Chi-squared test of homogeneity. For this analysis, the 2014 and 2015-cohorts were excluded as they did not have the full three-year follow-up time. The cumulative number of outpatient antimicrobial prescriptions per child was stratified into brackets < 1, 1–4, 5–9, 10–14 and ≥ 15 and the incidence risk ratio between VNEC and VEC was calculated for each bracket.

### Regression analysis

The Andersen-Gill extension of the Cox [29] regression model for repeated events was used to model the individual level data and allow for censored follow-up times. The model included age as the underlying measure of time to directly correct for age and was stratified allowing different baseline-hazards for each gender. The cumulative number of previous antimicrobial prescriptions was included as a covariate and modelled using a restricted cubic spline relationship. Finally, birth-cohort membership was included as a categorical variable. Robust sandwich variance estimates were included to account for correlation between successive observations of the same child. The model was used to estimate the hazard ratio (HR) of antimicrobial prescription between each of the study birth-cohorts and the reference birth-cohort. The vaccine impact of PHiD-CV10 in reducing outpatient antimicrobial prescriptions was then estimated as 1 – (the hazard ratio between the last vaccine eligible cohort and the reference cohort) × 100%.

The vaccine impact against each successive prescription was estimated by calculating the hazard ratio of antimicrobial prescription between VEC and VNEC for each number of previous prescriptions.

Finally, the mean number of antimicrobial prescriptions for each gender and vaccine-cohort was calculated as a function of age using the generalized Nelson-Aalen estimate [29]. This was used to estimate the absolute number of prevented antimicrobial prescriptions during the first seven years of the intervention by adding together the expected number of prescriptions per child using the VNEC estimate of the mean and subtracting the expected number of prescriptions per child using the VEC estimate of the mean. The absolute rate reduction was calculated by dividing this estimate with the number of person-years at-risk in the VEC.

A sub-analysis was performed to estimate the vaccine impact against AOM-associated antimicrobial prescriptions. The same regression methodology was applied to a subset of the prescriptions which were linked to a primary care physician visit with a diagnosis of AOM. Vaccine impact was similarly estimated as 1 – (the hazard ratio between the last vaccine eligible cohort and the reference cohort) × 100%.

## Results

### Population

Information was available for 53,218 children born from 2005 to 2015. Children who immigrated to Iceland (1892) were excluded from the analysis. An additional 756 children were excluded due to lack of information on gender or date-of-birth. The remaining 50,570 children were followed for a total of 140,429 person-years and accumulated 226,181 antimicrobial prescriptions. Of the children who had a full three-year follow-up time, the median number of prescriptions per child was 4 prescriptions (IQR 2–7, range 0–55). Person-years at-risk by age-group for children in the VNEC and VEC is shown in Table 3.

### Antimicrobial usage

First-line penicillins were the most commonly prescribed antimicrobials in 2005–2012 and represented between 41 and 47% of all prescriptions. In 2013, their use decreased to 32% and in 2014 and 2015 to 18%. In 2016, it increased to 35%. Concurrently, the use of second-line penicillins increased from 35 to 40% in 2005–2012, to 48%, 55%, and 54% in 2013, 2014 and 2015 respectively, before decreasing to 42% in 2016. The use of cephalosporins was 5.2–7.8% in 2005–2012 and increased to 10–15% in 2013–2016. Use of macrolides and other antimicrobials can be seen in Table 2.

**Table 2:** Number of prescription per calendar year for children < 3 years of age and the proportion of each antimicrobial class

Calendar year	Incidence of prescriptions per 100 person-years (n)	First line penicillins (%)	Second line penicillins (%)	First generation macrolides (%)	Second generation macrolides (%)	Cephalosporins (%)	Other (%)
2005	204 (25649)	41.41	37.92	1.48	6.55	5.37	7.26
2006	205 (26396)	40.34	39.57	1.27	6.22	5.36	7.24
2007	192 (25179)	44.97	36.80	1.60	6.39	5.16	5.08
2008	178 (24046)	46.74	35.22	0.20	6.37	5.91	5.57
2009	159 (22406)	46.41	37.16	0.05	5.51	6.33	4.55
2010	167 (24007)	43.71	38.55	0.02	5.54	7.02	5.17
2011	164 (23866)	44.70	37.92	0.03	5.91	7.47	3.98
2012	160 (22703)	43.45	39.01	0.01	6.92	7.77	2.83
2013	152 (21113)	32.10	48.08	0.02	6.56	10.03	3.20
2014	152 (20325)	18.48	55.46	0.01	6.60	14.53	4.92
2015	150 (19873)	18.49	53.91	0.06	7.25	14.95	5.34
2016	160 (20543)	35.28	41.68	0.04	5.52	12.91	4.57

### Crude incidence rate and incidence rate ratios

**Fig. 1:**
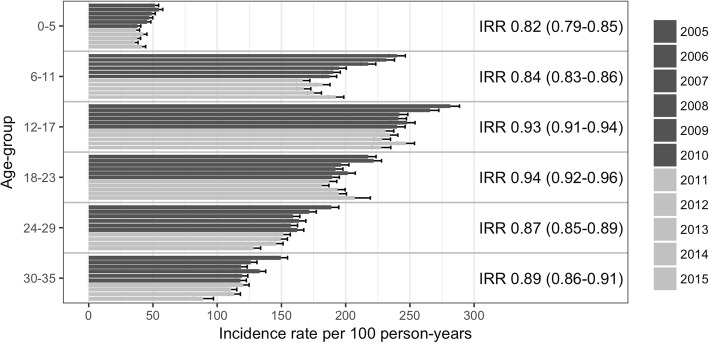
Incidence rate (IR) of antimicrobial prescriptions per 100 person-years for each birth-cohort for each of the six age groups as columns, blue for the vaccine non-eligible cohorts (NVEC, born 2005–2010) and red for the vaccine eligible cohorts (VEC, born 2011–2015). Error bars represent 95% confidence interval for IR. Incidence rate ratio (IRR) between the VNEC and VEC with 95% confidence intervals are displayed at the end of the columns

The overall crude rate of antimicrobial prescriptions for children < 3 years of age was significantly lower in the VEC than the VNEC, 150.3 and 167.6 antimicrobial prescriptions per 100 person-years respectively (IRR 0.90, 95%CI 0.89–0.91). The crude incidence per six-month age-groups are shown in Table 3. The crude incidence rate was highest among children 12–17 months of age in both VNEC and VEC, 247 and 233 prescriptions per 100 person-years respectively. The incidence rate of prescriptions decreased in all age-groups (IRR 0.82–0.94) with the largest decrease (IRR 0.82, 95%CI 0.79–0.85) in children < 6 months of age (Fig. 1).

**Table 3:** Incidence rate (IR) of all-cause and AOM-associated antimicrobial prescriptions per 100 person-years, comparing the Vaccine non-eligible cohorts (born 2005–2010, VNEC) to the Vaccine eligible cohorts (born: 2011–2015, VEC). ^a^Primary care data was only available until December 31st 2015. Therefore fewer person-years at-risk were available for the VEC when considering AOM-associated prescriptions and are as follows; 10,719; 9613; 8508; 7323; 6215 and 5083

Age-groups (months)	VNEC			VEC		
	IR (n of prescriptions)		Person-years at-risk	IR (n of prescriptions)		Person-years at-risk
	All cause	AOM-associated		All cause	AOM-associated	
< 6	47.0 (6816)	11.6 (1681)	14,491	39.1 (4338)	7.58 (813)	11,096^a^
6–11	206 (29,674)	69.1 (9931)	14,378	176 (19,447)	52.5 (5045)	11,024^a^
12–17	247 (35,226)	72.9 (10,372)	14,233	233 (24,588)	62.2 (5295)	10,566^a^
18–23	199 (28,008)	49.9 (7309)	14,096	189 (17,876)	46.6 (3411)	9460^a^
24–29	164 (22,843)	35.0 (4882)	13,965	144 (12,069)	30.3 (1882)	8374^a^
30–35	125 (17,259)	24.1 (3331)	13,848	112 (7218)	21.1 (1074)	7218^a^

The crude cumulative proportion of antimicrobial prescriptions at three years of age was 86.7% and 84.9% for the VEC and VNEC. A significantly larger proportion of children in the VEC had 0 or 1–4 prescriptions compared to children in the VNEC (incidence risk ratios 1.13, 95%CI 1.06–1.21 and 1.08, 95%CI 1.05–1.11 respectively). Concurrently, the proportion of children who filled ≥5 prescription decreased (Table 4).

**Table 4:** The proportion and 95% confidence interval for the vaccine non-eligible cohorts (VNEC, born: 2005–2010) and the vaccine eligible cohorts (VEC, born 2011–2013) that had filled 0, 1–4, 5–9, 10–14 and ≥ 15 prescriptions at 36 months of age. Only children with full 36 months follow-up were included

Number of prescriptions	Incidence proportion (%)		Incidence risk ratio (95%CI)
	VNEC	VEC	
0	11.4	13.2	1.16 (1.10–1.23)
1–4	43.7	47.3	1.08 (1.06–1.11)
5–9	31.6	29.1	0.92 (0.89–0.95)
10–14	9.8	7.5	0.77 (0.72–0.82)
≥15	3.5	2.9	0.83 (0.74–0.93)
Total	100	100	

**Fig. 2:**
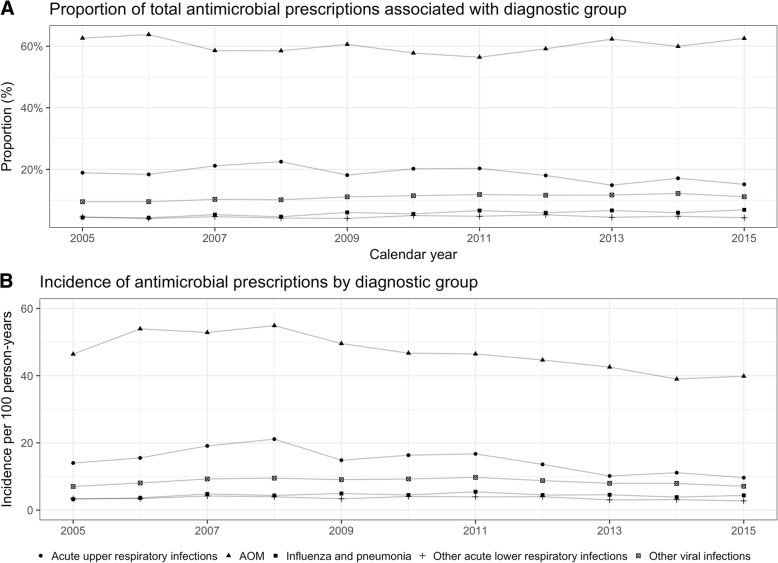
Antimicrobial prescriptions are linked to primary care visits if filled within three days of the index visit. Both graphs represent children < 36 months of age and are shown by calendar year. **a** represents the proportion of all antimicrobial prescriptions which were associated with a primary care visit due to a specific diagnostic group. **b** represents the incidence of antimicrobial prescriptions per 100 person-years which were associated with a diagnostic group

The proportion of visits due to AOM which resulted in an antimicrobial prescription increased gradually 2005–2015 from 57 to 64% (Additional file 1: Figure S1). The incidence rate of AOM-associated prescriptions decreased from a high of 54.9 prescriptions per 100 person-years in 2008 to 39.8 prescriptions per 100 person-years in 2015. The crude incidence rates of AOM-associated prescriptions by six-month age-groups are shown in Table 3. Concurrently, the proportion of AOM-associated antimicrobial prescriptions, out of all linked prescriptions remained stable between 59 and 66% (Fig. 2, Additional file 2: Table S1). Similarly, the incidence and proportion of visits due to acute upper respiratory infections which resulted in a antimicrobial prescription, increased from 2005 to 2008 after which it plateaued (Fig. 2 and Additional file 2: Table S1).

### Individual level regression modelling of all-cause antimicrobial prescriptions

**Fig. 3:**
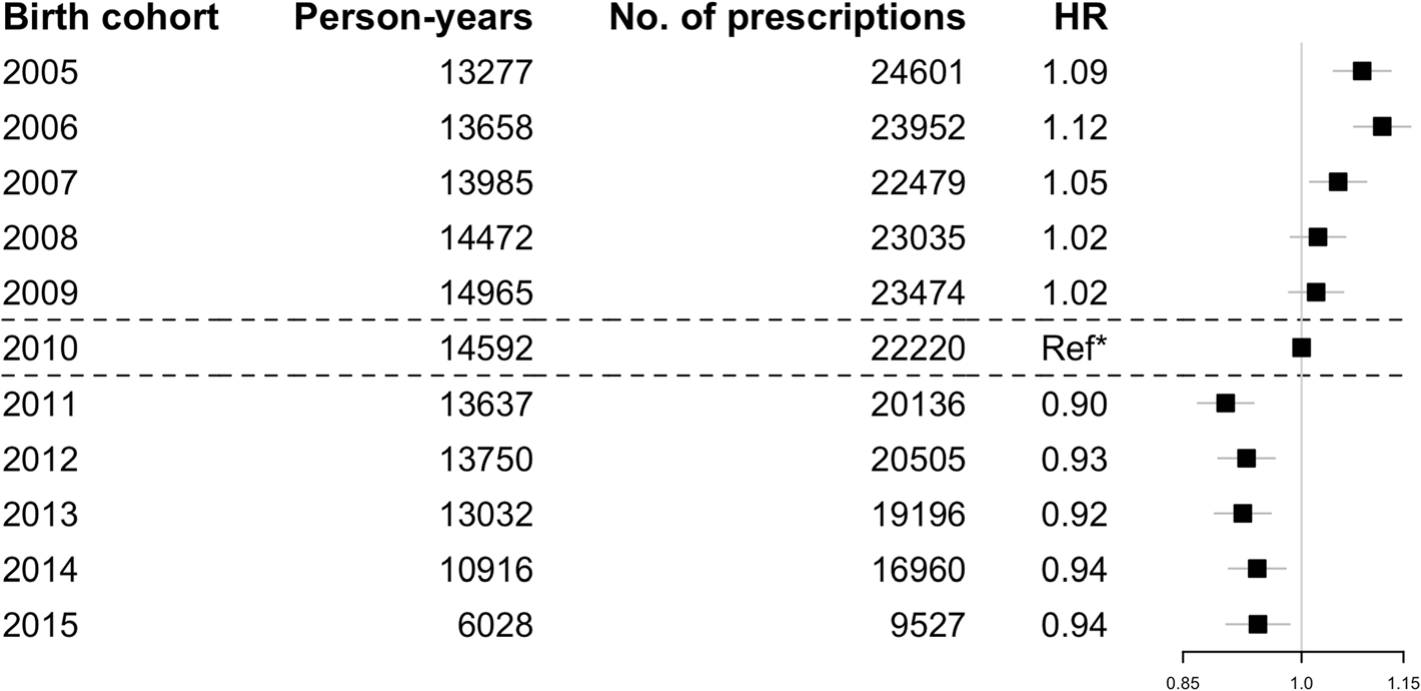
The hazard ratio (HR) from the cox-regression model for each of the study birth-cohorts compared to the last vaccine non-eligible birth-cohort (2010), the reference birth-cohort. Boxes represent point estimates for HR and lines the 95% confidence intervals

No significant deviation from model assumptions was noted in model diagnostic testing. The hazard of filling an antimicrobial prescription was significantly higher in the first three VNEC compared to the reference cohort (HR 1.08 95%CI 1.05–1.13, HR 1.12 95%CI 1.07–1.15) and HR 1.05 95%CI 1.01–1.09 respectively) while the hazard of birth-cohorts 2008 (HR 1.02 95%CI 0.98–1.06) and 2009 (HR:1.02 95%CI 0.98–1.06) did not differ from it significantly. Conversely, each vaccine-eligible birth-cohorts exhibited significantly lower hazard as compared to the reference cohort, with hazard ratios ranging from 0.90–0.94 (Fig. 3). The estimated PHiD-CV10 vaccine impact against all-cause antimicrobial consumption was 5.8% (95%CI 1.6–9.8%).

The hazard of receiving an additional antimicrobial prescription increased with each prescription, with the steepest increase following the first three prescriptions. The vaccine was associated with a significantly lower hazard of filling the first (HR 0.88, 95%CI 0.87–0.90), second (0.94 HR 0.92–0.96) and third (HR 0.97, 95%CI 0.95–0.99) prescriptions, but no significant difference was noted in the hazard of filling subsequent prescriptions.

**Fig. 4:**
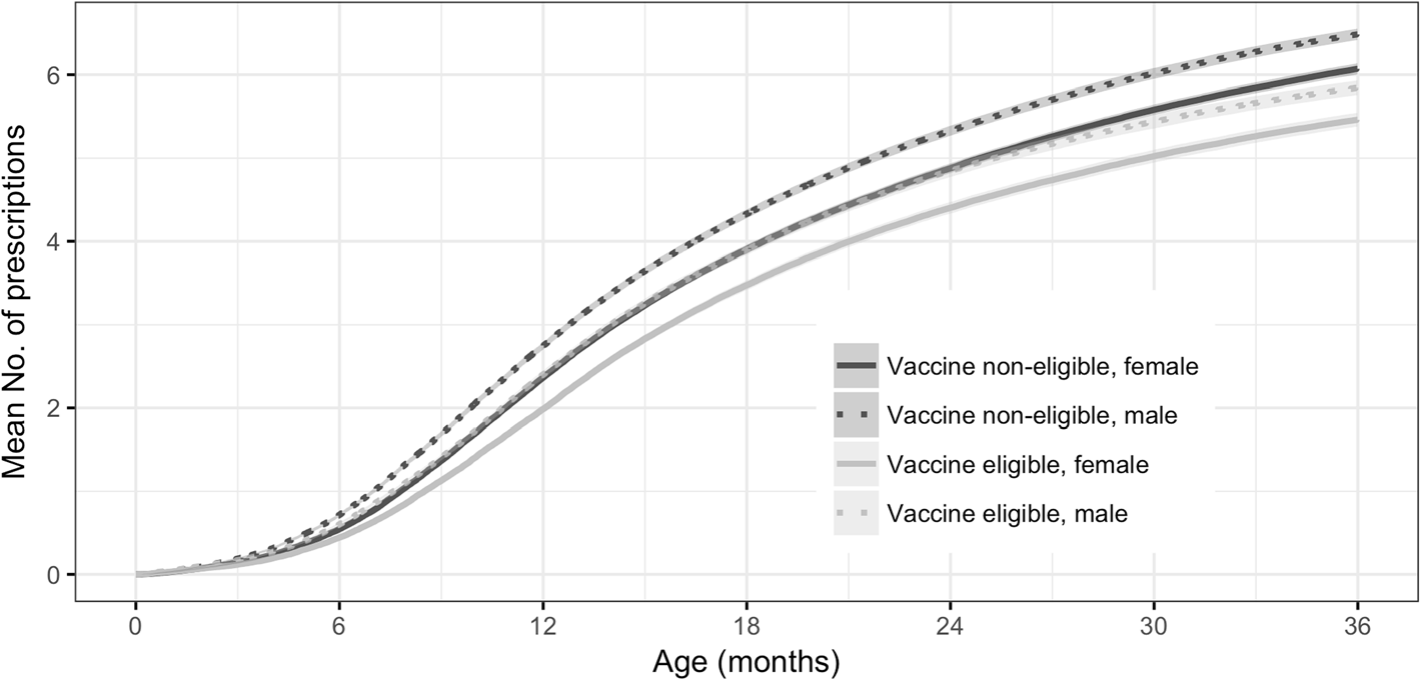
Expected mean number of prescription per child for the vaccine non-eligible cohorts (VNEC, born 2005–2010) and the vaccine eligible cohorts (VEC, born 2011–2015), for both genders from birth to third birthday. Lines represent point estimates and shaded area the 95% confidence intervals

The mean number of prescriptions in the first three years of life is displayed in Fig. 4. At 36 months of age, the mean number of prescriptions decreased from 6.07 (95%CI 6.00–6.14) to 5.46 (95%CI 5.38–5.55) among girls and from 6.48 (95%CI 6.42–6.55) to 5.84 (95%CI 5.76–5.93) among boys, in the VNEC and VEC respectively. The estimated absolute number of prevented prescriptions in the first six years of the intervention was 12,612 (95%CI 9471-15,752) with an absolute incidence rate reduction of 22.0 (95%CI 16.5–27.5) antimicrobial prescriptions per 100 person-years.

In the sub-analysis for AOM-associated antimicrobial prescriptions vaccine impact was calculated to be 21.8% (95%CI 11.5–30.9%), Additional file 3: Figure S2 shows HRs for each of the birth-cohorts compared to the 2010-reference cohort.

## Discussion

This population based study of 11 consecutive birth-cohorts followed until three years of age demonstrates a 5.8% reduction in all-cause outpatient antimicrobial prescriptions associated with the inclusion of PHiD-CV10 into the national paediatric vaccination program. We show that a relatively stable proportion of AOM visits result in antimicrobial prescriptions and that AOM is the most common indication for antimicrobial prescriptions in this age group. The impact of PHiD-CV10 on AOM-associated antimicrobial prescriptions was estimated 21.8%. Our group has previously demonstrated a 22% reduction in AOM visits following PHiD-CV10 introduction in Iceland [23]. Thus, a reduction in AOM episodes is a likely explanation for the reduction in all-cause antimicrobial prescriptions noted in this study. Age specific incidence rates of antimicrobial prescriptions were calculated for each birth-cohort and show a significant decrease in prescriptions in all age-groups following vaccine introduction. Also, the mean number of outpatient antimicrobial prescriptions for both the VNEC and VEC was estimated as a function of age and a significant decrease in both genders was demonstrated.

RCT have shown significant vaccine efficacy of PCVs against outpatient antimicrobial consumption, with outcomes ranging from 5.4% (95%CI 4.0–6.7) [25] to 8% (95%CI 1–14) [26] and 15% (95%CI 3–25) [24]. Our results are within the lower bounds of these findings. Though blinded RCT provide robust estimates of vaccine efficacy, they do so under artificial conditions. Parents and physicians may behave differently knowing that their actions are being observed and quantified by researchers, and this may reduce the incidence of inappropriate prescribing. Observational studies are valuable in demonstrating that vaccine impact against outpatient antimicrobial prescriptions can still be observed in true clinical settings. Several observational studies have previously assessed the change in antimicrobial prescriptions following PCV introduction, demonstrating a 20–42% decrease in AOM-associated prescriptions in children < 2 years of age [8, 21] and a 5–24% decrease in all-cause antimicrobial prescriptions in older children [30, 31]. These findings are in agreement with our results.

The present study has several strengths, chiefly in the inclusion of the whole population of children under three years of age. Because all Icelandic children in 11 consecutive birth-cohorts are included, the possibility of sampling bias is eliminated. In Iceland, antimicrobials for systemic use are exclusively available by physician prescription. When a prescription is dispensed at any pharmacy in Iceland, information about the prescription is sent electronically to the NPD. The prescription is linked to the individual child’s national identification number which allows observation of prescriptions by individual children regardless of location or time.

By using all-cause antimicrobial consumption as the outcome measure without attempting to exclude cases unlikely to be attributable to diseases caused by pneumococci, no positive bias is introduced through case ascertainment. The fact that antimicrobial consumption in Iceland is close to the average for European countries [32] further generalizes the results of the study.

The PHiD-CV10 was introduced in 2011 into the routine childhood vaccination program without a catch-up schedule. The uptake was immediately high with over 97% of each vaccine eligible birth-cohort receiving ≥2 doses [27]. Prior to the introduction of PHiD-CV10, no systematic vaccination against pneumococcus was in place. Between 0 and 1.9% of birth-cohorts 2005–2008 received ≥2 doses of PCV, and this proportion increased to 6.9% and 18.7% in birth-cohorts 2009 and 2010 [27]. The increase in vaccine uptake in birth-cohorts 2009 and 2010 is likely due to heightened awareness of the impending inclusion of PHiD-CV10 into the vaccination program, with many parents and caregivers opting to pay out-of-pocket for the vaccine. This clear differentiation in vaccine coverage between the vaccine non-eligible birth-cohorts and vaccine eligible birth-cohorts permits the use of the cohorts as a proxy for vaccination status.

Finally, as the data was identifiable to the individual, it allowed the use of more sophisticated survival analysis methods which are normally reserved for non-observational studies. This allowed for the complex interaction between age, gender and the number of previous antimicrobial prescriptions to be considered when estimating the vaccine impact. A crude incidence rate analysis was also performed (Fig. 1). Such an analysis necessitates the creation of discrete age-groups for comparison as the incidence of antimicrobial prescription varies by age and some of the VEC have censored follow-up time. The results of the individual level analysis demonstrate that the vaccine decreased the risk of the first, second and third antimicrobial prescriptions. However, the vaccine was not associated with a further decrease in risk in those who had already filled ≥4 prescriptions. This provides evidence for a cumulative effect and is congruent with the results of the crude incidence analysis where the difference is most evident in the older age-groups. Because age is discretized in the crude analysis, the cumulative effect is not estimated and the vaccine impact is less apparent. Using the individual level analysis, it was also possible to demonstrate the mean number of prescriptions by age, taking gender, vaccine eligibility and number of previous prescriptions into account. Interestingly, antimicrobial consumption was significantly higher in boys than girls. The mean number of prescriptions for boys decreased significantly following vaccine introduction, becoming the same as that of girls prior to the vaccination (Fig. 4). This gender difference may be attributed to the fact that AOM is more common in boys than girls [33].

When children in the VEC were stratified by vaccination status, rather than birth-cohort, an abnormally high proportion of children who neither visited a physician nor filled an outpatient antimicrobial prescription was concentrated among those who did not have a documented vaccine dose. This is likely due to either under documentation of administered vaccine doses or children emigrating from Iceland without an official change in legal residence. Children who are for any reason unable to experience the study event, i.e. fill an outpatient antimicrobial prescription, are for the same reason likely to be unable to have a documented administered vaccine dose. This precluded an analysis using individual vaccination status to directly estimate the vaccine impact.

A significant downward trend in antimicrobial consumption was noted during the pre-vaccination era, with the final VNEC having significantly lower consumption than birth-cohorts 2005–2007 and non-significantly less consumption than birth-cohorts 2008 and 2009. Several contributing factors may contribute to these trends. Firstly, as mentioned above, the vaccine-uptake in the 2010 birth-cohort was noticeably higher than for the previous birth-cohorts. Theoretically, this could result in underestimation of the vaccine impact. However, the hazard ratio of antimicrobial prescription between the 2008 birth-cohort, of which only 1.8% had received > 2 PCV doses, and the 2010 birth-cohort was non-significant (HR 1.02 95%CI 0.98–1.06) which suggests that the 18.7% vaccination coverage among children in the 2010 birth-cohort did not introduce significant negative bias to the vaccine impact estimate. Secondly, inappropriate prescribing may have decreased during the first half of the study period, after the Directorate of Health published new guidelines on diagnosis and treatment of AOM in 2009 [17]. However, as the majority of Icelandic doctors go overseas for specialist training (mostly Sweden, UK and USA), keeping up to date on respective guidelines and policy after returning back home to Iceland. This could result in gradual change in behaviour, starting prior to implementation, rather than sudden change A study comparing outpatient antimicrobial use in European countries for 2004 and 2009 using drug-specific quality indicators found that the quality of antimicrobial prescriptions in Iceland was generally high. The quality increased between 2004 and 2009, rising in the European rankings from 12th to 7th place (out of 32) [34]. This may indicate that fewer inappropriate prescriptions were written, thus reducing prescriptions in general. However, we did not see evidence of increasing quality of prescriptions during the study period using the parameters available to us, e.g. proportion of episodes receiving prescriptions and the proportion of narrow-spectrum antimicrobials use. To ensure that no positive bias was introduced due to decreases occurring prior to vaccine initiation the vaccine impact was calculated using the last vaccine non-eligible cohort as the reference, even though 18.7% of those children had received > 2 PCV doses.

As previously published by this study group, pneumococcal antimicrobial non-susceptibility is less common following the introduction of PHiD-CV10 [35], and fewer children have required parenteral therapy for AOM [36]. Under these circumstances, one would have expected the use of narrow spectrum antimicrobials to increase. However, the opposite was found. In 2013 antimicrobials classified as second-line penicillins became the most prevalent antimicrobial class prescribed in Iceland. The most likely explanation for this is the removal of the amoxicillin suspension from the general market in Iceland between August 2013 and August 2015. Physicians could still prescribe the amoxicillin suspension, but it required a written form that was generally considered a nuisance as compared to the electronic prescription form to which Icelandic physicians had grown accustomed. Instead of an increased use of other suspensions within the first-line penicillin class, such as phenoxymethylpenicillin, the use of second-line penicillins (amoxicillin/clavulanic acid) and cephalosporins increased. As amoxicillin/clavulanic acid has a higher rate of adverse reactions compared to first line penicillins [37, 38] the removal of amoxicillin may have resulted in a substantial burden on children and their parents. In addition, at the end of the study period and 18 months after the reintroduction of amoxicillin into the general market, its use was still lower than before its removal. This warrants further investigation.

## Conclusions

The introduction of PHiD-CV10 lead to reduced antimicrobial use in children, which was mostly due to reduced episodes of acute otitis media. This intervention therefore not only reduces disease burden but could also slow the spread of antimicrobial resistance.

## Additional files

Additional file 1: **Figure S1.** Proportion of primary care visits which resulted in a filled antimicrobial prescription by calendar year and by diagnostic group. (TIFF 8627 kb)

Additional file 2: **Table S1.** The proportion of linked antimicrobials associated with each diagnostic group by calendar year. Same data is presented in Fig. 2. (DOCX 33 kb)

Additional file 3: **Figure S2.** The hazard ratio (HR) for AOM-associated antimicrobial prescriptions from the cox-regression model for each of the study birth-cohorts compared to the last vaccine non-eligible birth-cohort (2010), the reference birth-cohort. Boxes represent point estimates for HR and lines the 95% confidence intervals. (TIFF 9621 kb)

## Abbreviations

AOM: Acute otitis media
ATC: Anatomical therapeutic chemical
HR: Hazard ratio
ICD-10: International Classification of Diseases, 10th Revision
IR: Incidence rates
IRR: Incidence rate ratios
NPD: National Prescription Database
PCV: Pneumococcal conjugate vaccine
PHiD-CV10: 10-valent pneumococcal non-typeable *Haemophilus influenzae* protein D-conjugate vaccine
RCT: Randomized controlled trials
URTI: Upper respiratory tract infections
VEC: Vaccine-eligible cohorts, VNEC: Vaccine non-eligible cohorts
