# Supplementary figures and images for: Impact of the 10-valent pneumococcal conjugate vaccine on antimicrobial prescriptions in young children: a whole population study
Source: BMC Infect Dis. 2018 Oct 4;18:505. doi: 10.1186/s12879-018-3416-y (PMC6172799; doi:10.1186/s12879-018-3416-y)

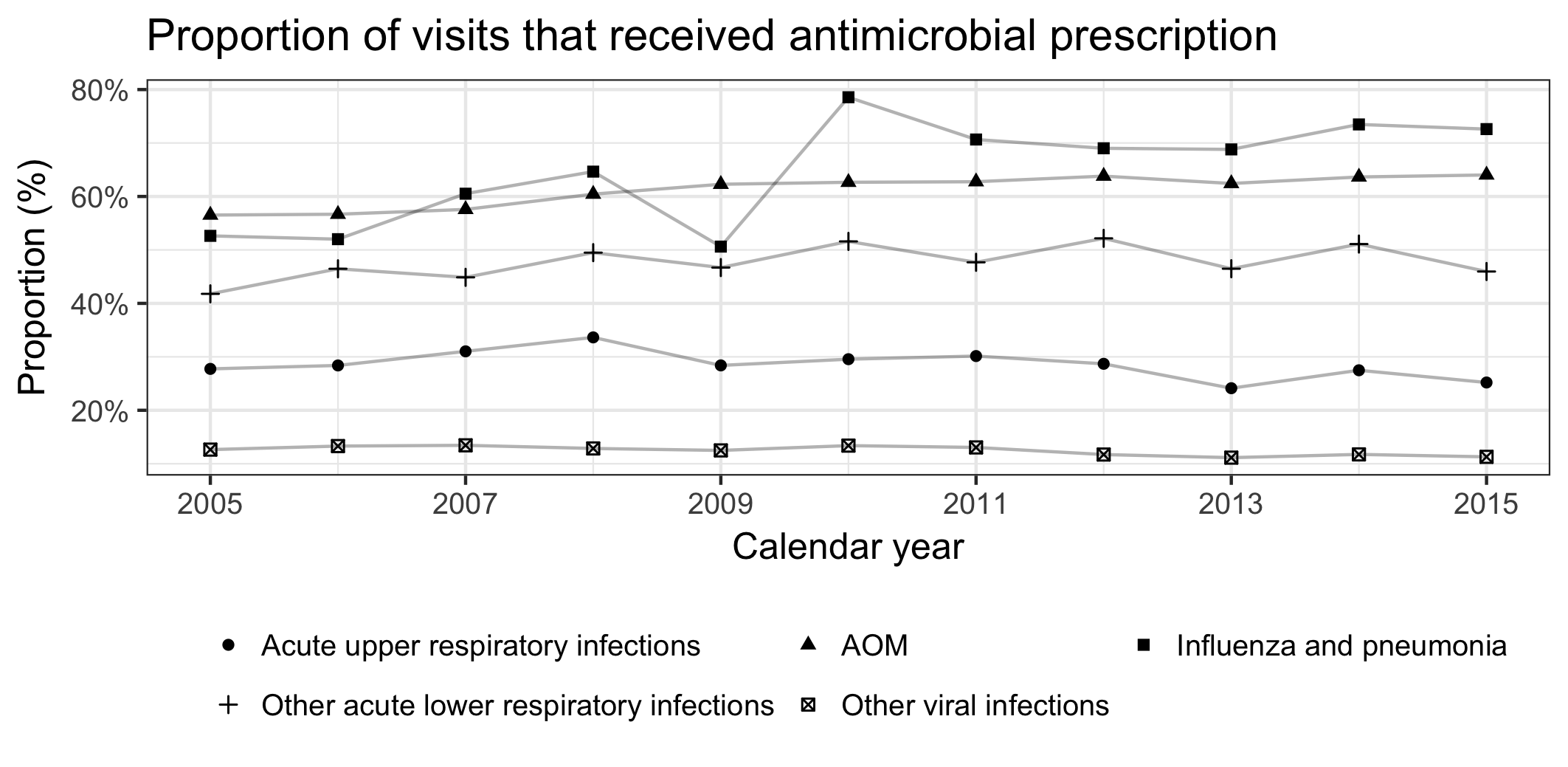

Supplement: Supplementary file 1 — Figure S1. Proportion of primary care visits which resulted in a filled antimicrobial prescription by calendar year and by diagnostic group. (TIFF 8627 kb) [file 12879_2018_3416_MOESM1_ESM.tiff]

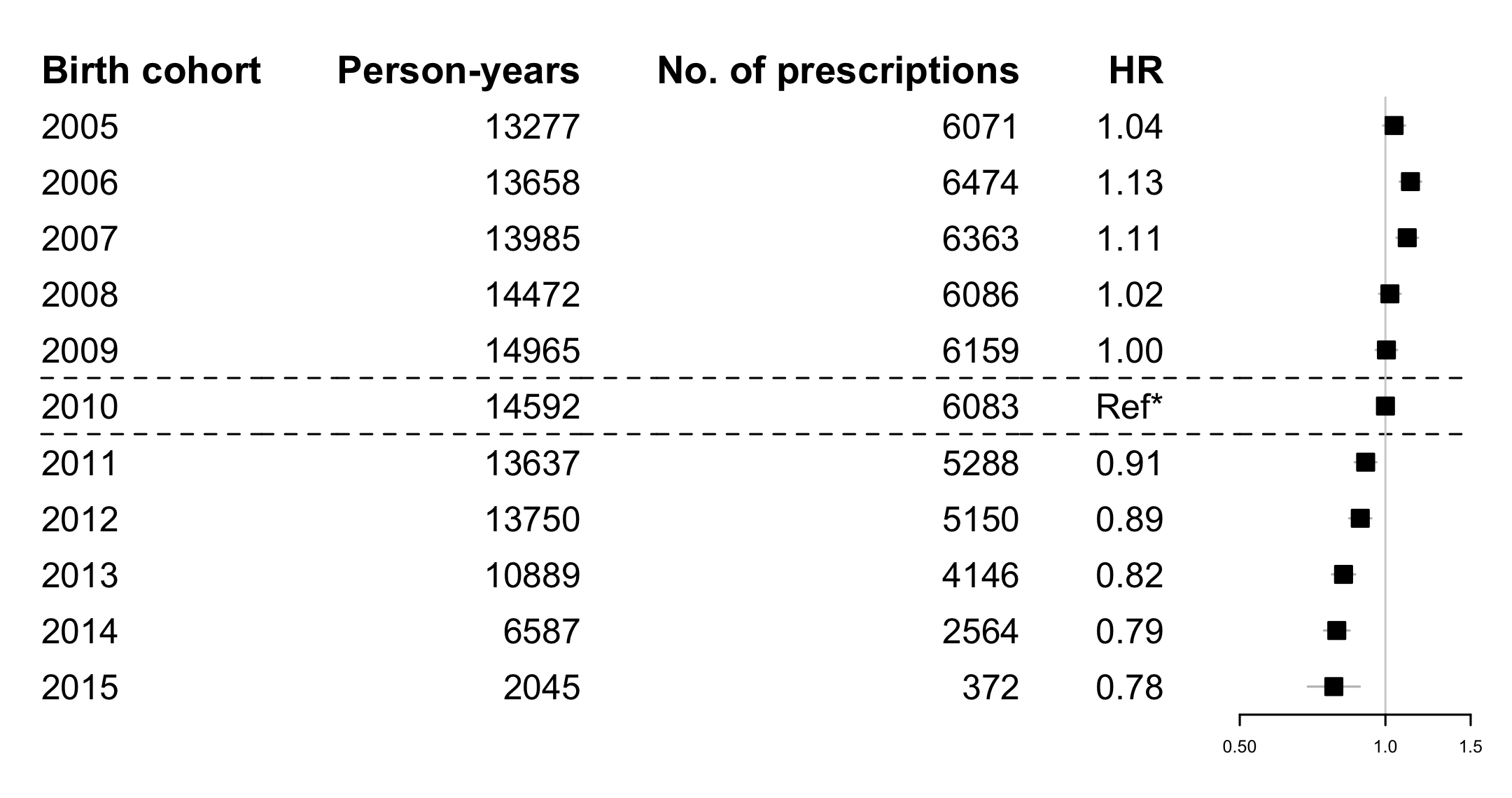

Supplement: Supplementary file 3 — Figure S2. The hazard ratio (HR) for AOM-associated antimicrobial prescriptions from the cox-regression model for each of the study birth-cohorts compared to the last vaccine non-eligible birth-cohort (2010), the reference birth-cohort. Boxes represent point estimates for HR and lines the 95% confidence intervals. (TIFF 9621 kb) [file 12879_2018_3416_MOESM3_ESM.tiff]
