# Supplementary material for: Impact of the 10-valent pneumococcal conjugate vaccine on antimicrobial prescriptions in young children: a whole population study
Source: BMC Infect Dis. 2018 Oct 4;18:505. doi: 10.1186/s12879-018-3416-y (PMC6172799; doi:10.1186/s12879-018-3416-y)
Supplement: Supplementary file 2 — Table S1. The proportion of linked antimicrobials associated with each diagnostic group by calendar year. Same data is presented in Fig. 2. (DOCX 33 kb) [file 12879_2018_3416_MOESM2_ESM.docx]

Table S1. The proportion of linked antimicrobials associated with each diagnostic group by calendar year. Same data is presented in Figure 2.

| Year | AURTI (%) | AOM (%) | Influenza & pneumonia (%) | Other ALRTI (%) | Other viral infections (%) |
| --- | --- | --- | --- | --- | --- |
| 2005 | 18.9 | 62.6 | 4.40 | 4.58 | 9.50 |
| 2006 | 18.4 | 63.8 | 4.29 | 4.06 | 9.53 |
| 2007 | 21.2 | 58.6 | 5.33 | 4.66 | 10.3 |
| 2008 | 22.5 | 58.5 | 4.64 | 4.22 | 10.1 |
| 2009 | 18.1 | 60.6 | 6.04 | 4.12 | 11.1 |
| 2010 | 20.2 | 57.8 | 5.55 | 5.01 | 11.5 |
| 2011 | 20.3 | 56.4 | 6.63 | 4.79 | 11.8 |
| 2012 | 18.0 | 59.1 | 5.93 | 5.27 | 11.6 |
| 2013 | 14.9 | 62.3 | 6.67 | 4.46 | 11.7 |
| 2014 | 17.1 | 59.9 | 5.95 | 4.81 | 12.2 |
| 2015 | 15.2 | 62.5 | 6.89 | 4.30 | 11.1 |
